# Effect of noisy galvanic vestibular stimulation in community-dwelling elderly people: a randomised controlled trial

**DOI:** 10.1186/s12984-018-0407-6

**Published:** 2018-07-03

**Authors:** Yasuto Inukai, Mitsuhiro Masaki, Naofumi Otsuru, Kei Saito, Shota Miyaguchi, Sho Kojima, Hideaki Onishi

**Affiliations:** 1 Department of Physical Therapy, Niigata University of Health and Welfare, 1398 Shimami-cho, Kita-ku, Niigata City, Niigata, Japan; 2 Institute for Human Movement and Medical Sciences, Niigata University of Health and Welfare, 1398 Shimami-cho, Kita-ku, Niigata City, Niigata, Japan

**Keywords:** Community-dwelling elderly people, Falls, Noisy galvanic vestibular stimulation, Centre of pressure, Postural sway

## Abstract

**Background:**

Balance disorders are a risk factor for falls in the elderly. Although noisy galvanic vestibular stimulation (nGVS) has been reported to improve balance in young people, randomised control trials targeting community-dwelling elderly people have not been conducted to date. We aimed to assess the influence of nGVS on COP sway in the open-eye standing posture among community-dwelling elderly people in a randomised controlled trial.

**Methods:**

A randomised controlled trial of 32 community-dwelling elderly people randomly assigned to control (sham stimulation) and an nGVS groups. All participants underwent centre of pressure (COP) sway measurements while standing with open eyes at baseline and during stimulation. The control group underwent sham stimulation and the nGVS group underwent noise stimulation (0.4 mA; 0.1–640 Hz).

**Results:**

In the nGVS group, sway path length, mediolateral mean velocity and anteroposterior mean velocity decreased during stimulation compared with baseline (*P* < 0.01). The effect of nGVS was large in participants with a high COP sway path length at baseline, but there was no significant difference in COP sway in the control group.

**Conclusions:**

We conclude that nGVS decreases the COP sway path length and mean velocity of community-dwelling elderly people when standing with open eyes. This suggests that nGVS could be effective for treating balance dysfunction in the elderly.

## Background

Falls are a leading cause of injury and death among the elderly and are a significant public health issue. It has been reported that one in three elderly people aged 65 years or older and half of those aged 80 years and older will fall once a year [1], with balance disorders being a major risk factor [2]. The visual, proprioceptive and vestibular sensory systems provide feedback from the environment and contribute to balance control by facilitating interaction with the external world [3, 4]. Among these, the vestibular system primarily functions to detect motion and head position. Specifically, three semicircular canals can perceive angular acceleration and velocity of the head, and the otolith organs (utricle and saccule) can sense linear acceleration of the head and head tilt [5]. However, vestibular system function declines with increasing age [6], and this can increase the risk of falls [7, 8].

To date, no effective treatment methods other than physical therapy have been established for the dysfunction of the vestibular system [9]. However, recently, noisy galvanic vestibular stimulation (nGVS) has shown some promise in this regard. This treatment acts by stimulating the vestibular organ with a weak noise current and has been shown to enhance vestibular perception and vestibulo-spinal reflex function [10, 11]. In a previous studies, nGVS was shown to enhance cognitive abilities in healthy subjects [12], to improve motor responsiveness in patients with central neurodegenerative disorders [13], and to improve gait parameters and standing balance in patients with vestibular disorders [14, 15]. Moreover, nGVS in the frequency band 0.1–640 Hz has been reported to improve postural sway in young subjects maintaining an open-eye standing posture and to produce a large stimulation effect in those with a long centre of pressure (COP) sway path [16]. In contrast, nGVS may decrease COP sway in the elderly [17]; however, because COP sway was measured in a closed-eye standing position on foam rubber in previous research, the influence on COP sway while on a firm surface remains unclear. The lack of a control group in the previous study also meant that we could not deny the effects of arousal, motor learning, and other factors than nGVS.

The purpose of this research was to clarify the effect of nGVS in the open-eye standing posture by performing a randomised controlled trial of community-dwelling elderly people. We also aimed to recognize individuals who responded to nGVS.

## Methods

### Subjects

We conducted a randomised controlled trial among community-dwelling elderly people. The inclusion criteria were that participants needed to be living independently, that they could maintain a standing position with their eyes open and legs together for 30 s without developing dizziness, and that they had no orthopedic or neurological disease. We excluded all potential participants who had previously undergone orthopedic surgery or who had neurological disease. Participants were randomly assigned to a control group or an nGVS group after being fully informed of the nature of the research and providing written informed consent. The study was performed in accordance with the Declaration of Helsinki and was approved by the ethics committee of Niigata University of Health and Welfare (17750e161007).

### Noisy galvanic vestibular stimulation

All nGVS was delivered using a DC-STIMULATOR PLUS (Eldith, NeuroConn GmbH, Ilmenau, Germany). Circular electrodes with 2.0 cm diameters were used as stimulating electrodes and applied to the mastoid process bilaterally. In the nGVS group, the stimulation intensity was 0.4 mA, and the stimulation frequency band was 0.1–640 Hz. Sham stimulation (0 mA) was performed in the control group.

### Measurement of COP

COP sway was measured for 30 s at 100 Hz in a standing position with eyes open and legs together, using a CFP400PA102RS (Leptrino, Japan). Participants were instructed to look at a mark 2 m ahead of them while standing. We calculated the average COP root mean square (RMS) area, the sway path length, the mediolateral (ML) mean velocity and the anteroposterior (AP) mean velocity [16].

### Clinical measures of postural stability

In addition to COP sway measurements, we evaluated clinical measures of postural stability, including the timed up and go (TUG) test and the one leg stance (OLS) test. For the TUG test, we observed and timed standing from a chair, walking 3 m, turning around, walking back to the chair and sitting down. The TUG time was the time, in seconds, that participants needed to complete the test. Longer times indicated worse balance and mobility [18]. The OLS test involved two trials of attempting to stand on one leg for 120 s, recording the maximum time. The OLS test can predict frailty in the community-dwelling elderly [19].

### Experimental procedures

**Fig. 1:**
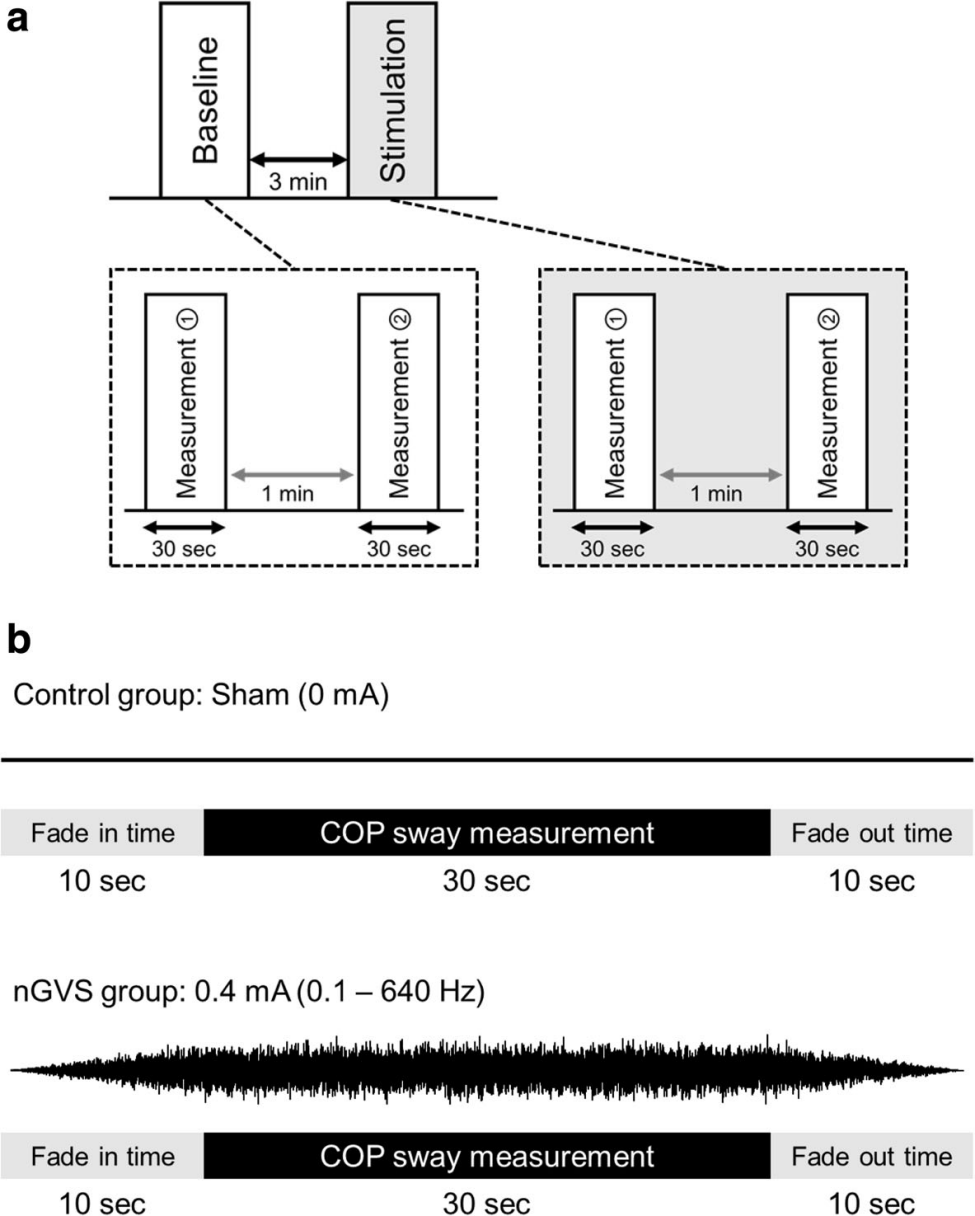
Overview of experimental design. **a**: Experimental procedures. Subject underwent COP measurements at the baseline and during sham stimulation or nGVS. Rest time was set to 1 min between measurements, but 3 min between the baseline and stimulation groups. **b**: The stimulation conditions for each group. Fade-in and fade-out times were both set to 10 s for nGVS. We performed COP measurement after the fade-in time. nGVS, noisy galvanic vestibular stimulation; COP, center of pressure

Participants underwent COP sway measurements twice without stimulation (baseline) and twice during stimulation. A rest time of 1 min was allowed between measurements at baseline and during stimulation, but a rest time of 3 min was allowed between the baseline and stimulation measurements. Noise stimulation (0.4 mA; 0.1–640 Hz) was applied to the nGVS group and sham stimulation (0 mA) was applied to the control group during the stimulation phases. Fade-in and fade-out times were both set to 10 s, and COP sway measurement was performed during stimulation (30 s), after the fade-in time (Fig. 1).

### Statistical analysis

IBM SPSS, Version 22 (IBM Corp., Armonk, NY, USA) was used for the statistical analyses, with the significance level set at 5%. Independent-sample Student *t*-tests were performed to evaluate the differences in characteristics, clinical measures of postural stability and COP sway measurement between the control and nGVS groups at baseline. Intraclass correlation coefficients (ICC [1, 2]) between each successive baseline and stimulation measurements were calculated to test the reliability of the results. The RMS area, sway path length, ML mean velocity and AP mean velocity at baseline and during stimulation were compared by two-way mixed-design analysis of variance (ANOVA), as follows: (time [baseline, stimulation]) × (group [control, nGVS]). When a significant difference was observed in the interaction, a post-hoc paired Student *t*-test test was applied. Moreover, Pearson’s product moment correlation coefficient was calculated for the sway path length between baseline and the stimulation effect. Furthermore, Pearson’s product moment correlation coefficient was calculated between the clinical measures of postural stability (TUG and OLS) and the stimulation effect. The stimulation effect was calculated for each group using the baseline value before each stimulation and the value during stimulation condition, as follows: (Δ stimulation effect = [baseline] – [stimulation]) [16].

## Results

We enrolled 32 community-dwelling elderly people (7 males and 25 females; mean age 75.8 ± 0.8 years) who were randomly assigned to a control group (3 males, 13 females, mean age 75.9 ± 1.1 years) and an nGVS group (4 males, 12 females, mean age 75.7 ± 1.3 years). No significant differences were found between the groups in their baseline characteristics, clinical measures of postural stability and COP sway measures (Table 1). The ICC for the RMS area was low (0.42–0.94) when compared with sway path length (0.89–0.93), ML mean velocity (0.87–0.91) and AP mean velocity (0.86–0.95) (Table 2).

**Table 1:** Comparison of participant details at baseline between the control and nGVS groups

		All (*n* = 32)	Control (*n* = 16)	nGVS (*n* = 16)	*P* value
Sex	Male	7	3	4	–
	Female	25	13	12	
Characteristics	Age (years)	75.8 ± 0.8	75.9 ± 1.1	75.7 ± 1.3	0.92
	Weight (kg)	55.0 ± 1.5	53.4 ± 2.3	56.7 ± 1.8	0.28
	Height (cm)	154.5 ± 1.3	153.2 ± 2.3	155.8 ± 1.8	0.34
Clinical measures of postural stability	TUG (s)	6.7 ± 0.2	6.5 ± 0.2	6.9 ± 0.3	0.40
	OLS (s)	60.2 ± 7.6	64.9 ± 10.8	55.4 ± 11.0	0.54
COP sway measures at baseline	RMS area (mm^2^)	224.5 ± 22.9	187.9 ± 17.2	261.1 ± 41.2	0.11
	Sway path length (mm)	785.6 ± 43.3	789.4 ± 65.2	781.7 ± 59.2	0.93
	ML mean velocity (mm/s)	16.2 ± 0.9	16.0 ± 1.3	16.5 ± 1.3	0.79
	AP mean velocity (mm/s)	16.4 ± 1.0	16.7 ± 1.5	16.1 ± 1.5	0.75

Characteristics (age, weight and height), clinical measures of postural stability (TUG and OLS) and COP sway measures were not significantly different between the control and *nGVS* groups at baseline. *AP* anteroposterior, *COP* centre of pressure, *ML*, mediolatera,; *nGVS* noisy galvanic vestibular stimulation, *OLS*, one leg stance test, *RMS*, root mean square*, TUG*, timed up and go test

**Table 2:** ICCs between the repeated measurements at baseline and during stimulation

	Control group				nGVS group			
	Baseline		Stimulation		Baseline		Stimulation	
	ICC	95% CI	ICC	95% CI	ICC	95% CI	ICC	95% CI
RMS area	0.42	−0.67–0.80	0.68	0.08–0.90	0.75	0.31–0.91	0.94	0.83–0.98
Sway path length	0.89	0.70–0.96	0.93	0.79–0.98	0.89	0.69–0.96	0.93	0.81–0.98
ML mean velocity	0.87	0.64–0.95	0.91	0.73–0.97	0.89	0.69–0.96	0.90	0.73–0.97
AP mean velocity	0.86	0.71–0.96	0.93	0.80–0.98	0.91	0.74–0.97	0.95	0.85–0.98

ICC = intraclass correlation coefficient (model 1, 2).Abbrevations: *95% CI* 95% confidence interval, *ANOVA* analysis of variance, *AP* anteroposterior, *ICC* Intraclass correlation coefficient, *ML* medio-lateral, *nGVS* noisy galvanic vestibular stimulation, *RMS* root mean square

**Fig. 2:**
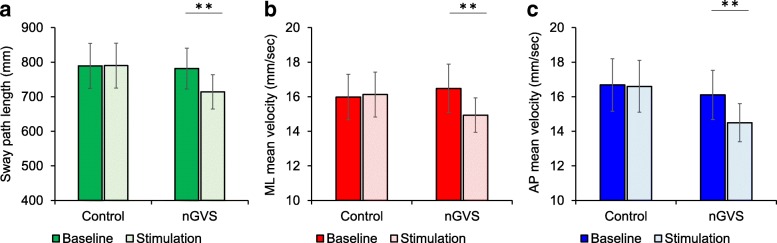
The effect of nGVS in the control and intervention groups. Bar charts of **a** sway path length, **b** ML mean velocity and **c** AP mean velocity (error bars indicate standard error; **p < 0.01)

**Fig. 3:**
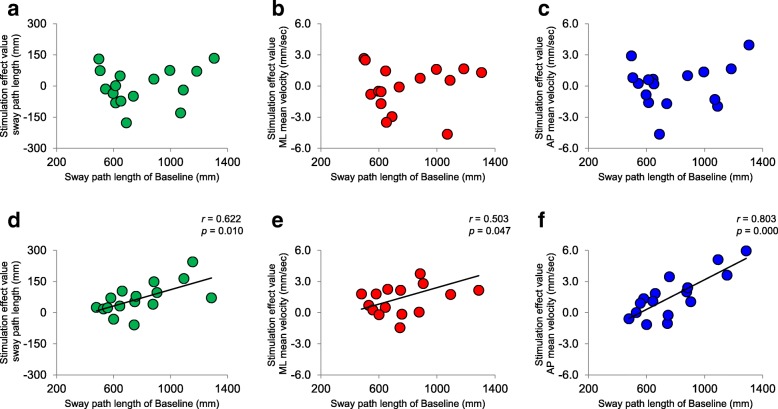
Scatter diagram of Baseline vs. Stimulation effect. **a** Sway path length, **b** ML mean velocity and **c** AP mean velocity in the control group. **d** Sway path length, **e** ML mean velocity and **f** AP mean velocity in the nGVS group. Significant correlations were found for all nGVS results. AP, anteroposterior; ML, mediolateral; nGVS, noisy galvanic vestibular stimulation

Table 3 shows the RMS area, sway path length, ML mean velocity and AP mean velocity for the control group and nGVS groups, together with the results of two-way mixed-design ANOVA. Sway path length and AP mean velocity revealed a significant main effect for time and for the interaction between time and group. ML mean velocity also had a significant interaction (time × group), but the RMS area had no significant interaction or main effect. Post-hoc analysis in the nGVS group revealed that sway path length (− 7.7 ± 1.9%), ML mean velocity (− 8.5 ± 2.4%) and AP mean velocity (− 8.3 ± 2.6%) were significantly decreased by stimulation compared with baseline (mean ± standard error) (Table 3 and Fig. 2). However, in the control group, no significant differences were found when comparing the results of stimulation with the baseline findings (sway path length: 0.2 ± 3.1%, ML mean velocity: 0.6 ± 4.0%, AP mean velocity: 0.4 ± 3.4%). Although there were significant correlations between the sway path length at baseline and the sway path length, ML mean velocity and AP mean velocity during stimulation effects in nGVS group, there were no such correlations in the control group (Fig. 3). In both the groups, there was no significant correlation between the clinical measures of postural stability (TUG and OLS) and stimulation effect (*p* > 0.05).

**Table 3:** COP sway (mean ± standard error) at baseline and during intervention in the control and nGVS groups and the results of two-way mixed-design ANOVA

Value of COP sway measurement	Control group			nGVS group		
	Baseline	Stimulation	*P* value	Baseline	Stimulation	*P* value
RMS area (mm^2^)	187.9 ± 17.2	182.2 ± 22.1	0.708	261.1 ± 41.2	244.9 ± 42.8	0.570
Sway path length (mm)	789.4 ± 65.2	790.3 ± 65.0	0.970	781.7 ± 59.2	714.3 ± 49.8	0.003
ML mean velocity (mm/s)	16.0 ± 1.3	16.1 ± 1.4	0.801	16.5 ± 1.3	14.9 ± 1.0	0.005
AP mean velocity (mm/s)	16.7 ± 1.5	16.6 ± 1.4	0.858	16.1 ± 1.5	14.5 ± 1.1	0.007
Two-way mixed-design ANOVA	Time		Group		Time × Group	
	*F* value	*P* value	*F* value	*P* value	*F* value	*P* value
RMS area	0.479 (1,30)	0.494	2.419 (1,30)	0.130	0.111 (1,30)	0.741
Sway path length	5.232 (1,30)	0.029	0.249 (1,30)	0.622	5.503 (1,30)	0.026
ML mean velocity	3.882 (1,30)	0.058	0.038 (1,30)	0.847	5.549 (1,30)	0.025
AP mean velocity	5.457 (1,30)	0.026	0.476 (1,30)	0.496	4.328 (1,30)	0.046

*COP* sway is reported as mean ± standard error, *AP* anteroposterior; *ANOVA* analysis of variance, *COP*, centre of pressure, *ML*, mediolateral, *nGVS*, noisy galvanic vestibular stimulation, *RMS* root mean square

## Discussion

We found significant decreases from baseline in sway path length, ML mean velocity and AP mean velocity among participants who underwent nGVS. By contrast, there was no significant difference between the baseline and stimulation data for the control group. Furthermore, the stimulation effect of nGVS was prominent in participants with high sway path length values at baseline. Notably, the significant decreases in sway path length, ML mean velocity and AP mean velocity in the intervention group were also associated with high ICC_(1,2)_ values (range 0.8–0.95), indicating that this result has high reliability [20].

Studies have shown that patients with vestibular disorders have increased COP sway path lengths and mean velocities [21] and that nGVS can enhance the function of vestibular afferents [22]. These findings suggest that the improved function of vestibular afferents may have reduced COP sway observed in this study. The rationale for these ameliorating effects of nGVS is considered to be stochastic resonance [22], a phenomenon where a signal that is too weak to exceed a given threshold is amplified by adding noise. Stochastic resonance appears to enhance information processing in the sensory system [23, 24]. Recently, nGVS has been reported to sufficiently lower the vestibulospinal reflex threshold to enable people to sense and process usually unrecognised subthreshold vestibular signals, thereby helping to decrease postural sway [11]. Furthermore, the afferent vestibular excitation induced by GVS, using direct current passes through the vestibular nucleus of the brainstem and vestibular thalamus, can activate brain areas associated multisensory input (areas 2, 3a/b and 7 and the parieto-insula vestibular cortex) [25]. Alternating-current GVS can also activate areas involved in processing vestibular information for head and body orientation in space (i.e. the supramarginal gyrus, posterolateral thalamus, cerebellar vermis, posterior insula and hippocampus) [26]. Even in nGVS, brain rhythms in a wide brain area have been reported to be modulated, and the involvement of stochastic resonance has been proposed [27]. The activation of cortical areas involved in multisensory input, including vestibular information, may be involved in the reduction of postural sway during nGVS.

We consider that vestibular afferent function was enhanced by nGVS in this study, and that postural sway decreased because of activation of the cortical region involved in vestibulospinal and vestibular sensory input. Furthermore, we found that nGVS was more effective for participants who had a high sway path lengths at baseline. Given that no such correlation was observed in the control group, we consider this a characteristic effect of nGVS. This is an important finding because sway path length is an independent predictor of falls in the elderly, with values known to be higher in fallers than in non-fallers [28, 29]. Similarly, it has been reported that ML and AP mean velocities are increased in fallers [30–32]. These support the argument that nGVS could be an effective treatment for appropriately screened elderly patients with balance disorders.

Interestingly, we found no change in the RMS area; but, the reliability of the sway area has been reported to be less than that of the mean velocity [33]. In addition, we found that the RMS area showed a low ICC, and this poor reliability may have affected our results. However, previous studies have shown that the sway area decreases during nGVS [10, 14]. In this study, nGVS was performed at a constant intensity (0.4 mA), whereas in previous studies it has been performed at intensities that were optimised for each subject. The lack of decrease in the RMS area could be explained by the difference in stimulus intensity settings. In addition, we used a stimulation frequency band of 0.1–640 Hz on the basis of a previous study [16, 34–37] although whether this band is optimal for nGVS remain unknown. The optimum stimulation condition (stimulation intensity and frequency band) for nGVS should be elucidated in future studies.

Further, although there was a significant correlation between the baseline COP sway path length and stimulation effect, there was no correlation between clinical measures of postural stability (TUG and OLS) and stimulation effect. In this study, we recruited only those subjects who lived independently, which may have resulted in the differences in clinical measures of postural stability being small among individuals. This, in turn, may have accounted for the failure to identify a correlation with irritation effect. In future research, we believe that including a larger cohort of elderly people with different balance disorders will help to resolve this issue.

## Conclusion

In conclusion, we have shown that nGVS decreases COP sway in a community-dwelling elderly population, producing a large stimulation effect in those with high COP sway path lengths in open-eyed standing who are at high risk of falls. However, we did not assess the effects of nGVS in fragile elderly patients who suffer from repeat falls and crucially did not determine whether nGVS decreases the incidence of falls. Although our data suggest that nGVS could be effective at preventing falls in the elderly, future research is needed to look at these remaining issues.
